# Type I Interferon Elevates Co-Regulatory Receptor Expression on CMV- and EBV-Specific CD8 T Cells in Chronic Hepatitis C

**DOI:** 10.3389/fimmu.2015.00270

**Published:** 2015-06-10

**Authors:** Solomon Owusu Sekyere, Pothakamuri Venkata Suneetha, Svenja Hardtke, Christine Susanne Falk, Julia Hengst, Michael Peter Manns, Markus Cornberg, Heiner Wedemeyer, Verena Schlaphoff

**Affiliations:** ^1^Department of Gastroenterology, Hepatology and Endocrinology, Hannover Medical School, Hannover, Germany; ^2^TTU-Hepatitis, TTU-IICH, German Center for Infectious Diseases (DZIF), Hannover-Braunschweig, Germany; ^3^Institute of Transplantation Immunology (IFB-Tx), Hannover Medical School, Hannover, Germany

**Keywords:** hepatitis C, IFN-alpha, cytokines, co-regulation, T cell regulation, PD-1, TIM-3, 2B4

## Abstract

Hepatitis C virus (HCV) readily sets up persistence in a large fraction of infected hosts. Mounting epidemiological and immunological evidence suggest that HCV’s persistence could influence immune responses toward unrelated pathogens and vaccines. Nonetheless, the fundamental contribution of the inflammatory milieu during persistent HCV infection in impacting immune cells specific for common pathogens such as CMV and EBV has not been fully studied. As the co-regulatory receptors PD-1, Tim-3, and 2B4 have all been shown to be vital in regulating CD8^+^ T cell function, we assessed their expression on CMV/EBV-specific CD8^+^ T cells from patients with chronic hepatitis C (CHC) and healthy controls *ex vivo* and upon stimulation with virus-specific peptides *in vitro*. Total and CMV/EBV-specific CD8^+^ T cells expressing PD-1, Tim-3, and 2B4 were highly enriched in patients with CHC compared to healthy individuals *ex vivo*. *In vitro* peptide stimulation further potentiated the differential co-regulatory receptor expression of PD-1, Tim-3, and 2B4, which then culminated in an enhanced functionality of CMV/EBV-specific CD8^+^ T cells in CHC patients. Comprehensively analyzing plasma cytokines between the two cohorts, we observed that not only was IFNα-2a dominant among 21 other inflammatory mediators elevated in CHC patients but it also correlated with PD-1 and Tim-3 expressions *ex vivo*. Importantly, IFNα-2a further caused upregulation of these markers upon *in vitro* peptide stimulation. Finally, we could prospectively study patients receiving novel IFN-free antiviral therapy. Here, we observed that treatment-induced clearance of HCV resulted in a partial reversion of the phenotype of CMV/EBV-specific CD8^+^ T cells in patients with CHC. These data reveal an alteration of the plasma concentrations of IFNα-2a together with other inflammatory mediators during CHC, which appeared to pervasively influence co-regulatory receptor expression on CMV/EBV-specific CD8^+^ T cells.

## Introduction

Chronic diseases caused by various pathogens impact a large proportion of the global population. Recent updated global estimates indicate that persistent infections with hepatitis C virus (HCV) alone affect about 80 million infected individuals (1). Like for many other viruses, antiviral T cell responses are particularly critical in limiting persistent infections and conferring lifelong protective immunity to viral re-exposures (2). In the majority of patients with chronic hepatitis C (CHC), however, viral clearance often fails as a result of an exhausted cytotoxic T cell response mediated by several co-regulatory receptors such as PD-1, CTLA-4, Tim-3, and 2B4 (3). Despite being functionally exhausted, T cell responses during persistent HCV infection remain strong enough to cause ongoing hepatocyte destruction (4). In the wake of the tissue pathology that ensues, several intrahepatic immune-modulatory cytokines (5) and chemokines (6) are induced.

Besides causing chronic intrahepatic inflammation and tissue destruction, persistent HCV infection has been associated with a plethora of extra-hepatic diseases, including autoimmune perturbations, lymphoproliferative disorders, diabetes, as well as neurological complications (7). Accumulating epidemiological evidence further suggests that persistent HCV infection might impact the incidence as well as immune responses toward unrelated pathogens and vaccines. Particularly, immune responses to vaccines against hepatitis A (8, 9), hepatitis B (10, 11), and influenza A (12) viruses are reduced in subjects with CHC. Similar to what pertains in HCV-related neuropathy (13), it is conceivable that the inflammatory mediators induced during persistent HCV infection contribute to influencing immune responses to other microbes. Indeed, an altered cytokine milieu was implicated in the substantial skewing of T cell differentiation, proliferation, and effector function observed in different settings of persistent virus and parasitic infections in both animal models and human diseases (11, 14, 15). Importantly, the impact of persistent infections transcends those that are pathogen specific as they may also have a global influence on the phenotype of the bulk T cell population (16). In spite of all these strides, the contribution of the inflammatory milieu during persistent HCV infection in impacting the phenotype and functional attributes of antigen-specific CD8^+^ T cell responses toward common pathogens such as CMV and EBV is not yet fully understood. So far, the limited data available on this subject are conflicting. While some reports suggest that persistent HCV infection may cause pervasive T cell intrinsic changes, which influence the phenotype of circulating CMV-specific CD8^+^ T cells (16, 17), others show otherwise (18).

In essence, we here evaluated whether persistent HCV infection may influence the phenotype and functionality of CMV/EBV-specific CD8^+^ T cells through distinct co-regulatory receptor molecules. Our results reveal a profound HCV-induced alteration of various inflammatory mediators, which appeared to influence the frequency, phenotype, and *in vitro* functionality of both total and virus-specific CD8^+^ T cells of CMV and EBV. Importantly, these effects normalized upon treatment-induced HCV clearance.

## Materials and Methods

### Study subjects

A total of 154 subjects participated in this study. This included 76 patients with CHC recruited from the liver outpatient clinic and 78 healthy individuals comprising blood donors from the internal blood donation center or healthy employees of the Hannover Medical School undergoing routine check-ups. All patients were anti-HIV and HBsAg negative and had no evidence of active CMV or EBV disease. Of the patients with CHC, 69 had not received any prior antiviral treatment, while 7 had received IFN-free antiviral therapy with sofosbuvir + ribavirin for 12–24 weeks. Untreated CHC patients had a compensated liver disease (13% cirrhosis) and a median viral load, ALT and AST of 1.2 × 10^6^ IU/ml, 71.5, and 54.5 U/l, respectively. The seven patients who had received sofosbuvir therapy had cleared the virus at end of therapy, developed a sustained virologic response, and were non-cirrhotic and normalized ALT and AST levels. For these patients, blood samples were collected at baseline (i.e., before therapy) and 12 weeks after end of therapy (FuW12). Patient cohorts and characteristics are summarized in Table 1. The calculation of the absolute cell numbers of CMV- and EBV-specific CD8^+^ T cells from patients with sofosbuvir treatment was performed using the frequencies of multimer-positive CD8^+^ T cells referring to the lymphocyte gate (frequency of lymphocytes) obtained by FACS analysis. The total lymphocyte counts obtained from clinical hematological diagnostics were used to calculate the absolute cell counts together with the FACS frequencies obtained.

**Table 1:** **Clinical characteristics of study cohorts**.

Parameter	Chronic HCV (no therapy)	Chronic HCV (sofosbuvir-treated)	Healthy
Time point		Baseline	
Number of subjects (*n*)	69	7	90
Number of HLA-A2 positive patients	61	7	67
Male sex – no. (%)	36 (52)	3 (43)	N/A
Age (years)			
Median	49	57	N/A
Range	22–80	53–67	N/A
HCV RNA – (IU/ml)			
Median	1.2 × 10^6^	730,000	N/A
Range	8.6 × 10^4^–9.8 × 10^6^	1.8 × 10^5^–1.1 × 10^7^	N/A
≥8 × 105 IU/ml – no./total no. (%)	39/63 (62)	3/7 (43)	N/A
Unknown	6/69 (9)	N/A	N/A
Undetectable	6/69 (9)	N/A	N/A
Alanine aminotransferase – (U/l)			
Median	71.5	67	N/A
Range	10–311	28–224	N/A
Above median – no./total no. (%)	34/68 (50)	3/7 (43)	N/A
Unknown	1/69 (1.5)	N/A	N/A
Aspartate aminotransferase – (U/l)			
Median	54.5	54	N/A
Range	22–279	27–118	N/A
Above median – no./total no. (%)	34/68 (50)	3/7 (43)	N/A
Unknown	1/69 (1.5)	N/A	N/A
HCV genotype – no. (%)			
1	50 (82)	0 (0)	N/A
2	2 (3)	1 (14)	N/A
3	9 (15)	5 (72)	N/A
5	0 (0)	1 (14)	N/A
Not determined	8 (12)	0 (0)	N/A

*N/A, not applicable*.

### Isolation and storage of PBMC and blood plasma samples

Peripheral blood mononuclear cells (PBMCs) were isolated from fresh blood samples using standard Ficoll density gradient centrifugation. Cells were cryopreserved in freezing medium consisting of 30% RPMI-1640 medium (Invitrogen, Karlsruhe, Germany), 60% fetal bovine serum (PAA, Pasching, Austria), and 10% DMSO (Sigma-Aldrich, Munich, Germany). Blood plasma sample collection was done from CPDA-treated blood samples and stored at −20°C.

### Peptides, HLA class I multimers, and recombinant proteins

Antigenic HLA-A*0201 restricted CMV-specific pp65_495–504_ (NLVPMVATV) and EBV-specific BMLF1_259–267_ (GLCTLVAML) peptides were purchased from ProImmune Ltd. (Oxford, UK). Peptides were dissolved in endotoxin-free DMSO (Sigma-Aldrich, Munich, Germany) and had a purity of >98%. Corresponding PE-labeled MHC class I CMV tetramer as well as EBV Dextramer were obtained from Beckman Coulter Inc. (Fullerton, CA, USA) and Immudex (Copenhagen, Denmark), respectively. For *in vitro* stimulations, PEG–IFN-alfa-2a (Pegasys; Roche) was used. Additionally, lyophilized recombinant human ICAM-1 and VCAM-1 were obtained from PeproTech Inc. (Rocky Hill, NJ, USA) and reconstituted in 0.1% bovine serum albumin solution according to the manufacturer’s instructions.

### Fluorescent antibodies and phenotypical staining

The following mouse anti-human monoclonal antibodies (MAb) were used: anti-PD1/clone EH12.2H7 (BioLegend Inc., San Diego, CA, USA), anti-2B4/clone C1.7 (Beckman Coulter, Fullerton, CA, USA) as wells as anti-Tim3/clone 344823, and anti-human IFNγ/clone 25723 (R&D Systems, Minneapolis, MN, USA). Anti-CD14/clone M5E2, anti-CD19/clone 5J25C1, anti-CD56/clone B159, anti-CD107b/clone H4B4, anti-TNF/clone MAb11, anti-CD8/clone SK1, and mouse IgG isotype controls were all obtained from BD Pharmingen (Becton Dickinson, Heidelberg, Germany). Tetramer and antibody stainings were performed as detailed previously (19). Cells were acquired using a BD FACSCanto II flow cytometer (Becton Dickinson, Heidelberg, Germany) and analyzed with FlowJo Software (TreeStar Inc., San Diego, CA, USA). A multimer-specific CD8^+^ T cell population was considered detectable if the frequency was at least 0.1% of total CD8^+^ T cells. Exclusion of unspecific events was done using a dump channel (CD14^+^CD19^+^CD56^+^). Respective FMO controls were included to enable gating of cell populations.

### *In vitro* cellular stimulation

Peripheral blood mononuclear cells were thawed and plated at 3–4 × 10^5^ cells/well in 96-well *U*-bottomed plates (Sarstedt GmbH, Nümbrecht, Germany). Respective CMV- or EBV-specific peptides at optimal concentrations (CMV: 1 μg/ml, EBV: 0.5 μg/ml) were added to stimulate the cells. Conditions of unstimulated cells were included as negative controls. All cultures were kept in AB-Medium (RPMI-1640), 10% heat-inactivated human AB serum (PAN Biotech GmbH, Germany), 1% non-essential amino acids, 1% sodium pyruvate, 1% penicillin/streptomycin, 0.5% HEPES buffer (GIBCO), and supplemented with 5 IU/ml fresh human recombinant IL-2 (Invitrogen, Karlsruhe, Germany) at day 3 and 7 during culture. For the cytokine/chemokine stimulation assay, PBMCs were stimulated with 10, 100, or 1000 ng/ml of ICAM-1, VCAM-1, or IFNα-2a in the presence or absence of virus-specific peptides. In all cases, cells were incubated at 37°C and 5% CO_2_ for 10 days.

### Intracellular cytokine staining and CD107a degranulation assay

After 10 days *in vitro* stimulation, PBMCs were resuspended in fresh AB medium and incubated in the presence of optimal concentrations of virus-specific peptides as described above. Brefeldin A was added after 1 h and cells incubated for additional 5 h. Surface staining including CD107 was carried out before intracellular staining for TNF and IFNγ was performed as described (19).

### Quantification of plasma cytokines and chemokines

The plasma concentrations of 50 cytokines and chemokines in patients with CHC and healthy controls as well as CHC patients treated with sofosbuvir were measured using the Luminex-based multiplex technology (Bio-Plex Pro Human Cytokine Panel, Bio-Rad, Hercules, CA, USA). The assay was performed as per the manufacturer’s instructions. Briefly, lyophilized cytokine/chemokine standards were resuspended in standard diluents and a series of serial dilutions performed in order to generate standard curves for each cytokine/chemokine. Fifty microliters of the resuspended standard cytokine/chemokine or plasma samples were pipetted and incubated for 30 min at RT with a mixture of beads having specificity for each cytokine or chemokine. The beads were washed several times with wash buffer. Biotinylated secondary antibody mixture was added and incubated for 30 min at RT, washed three times, before staining with streptavidin-conjugated R-phycoerythrin (SAPE). Beads were washed three times and resuspended in 125 μl assay buffer, acquired and analyzed using the BioPlex Manager 6.0 software. Our internal standard operating procedure for data analysis is available upon request.

### Statistical analyses

Data were analyzed using GraphPad Prism v6.0b (GraphPad software, La Jolla, CA, USA). Quantitative comparisons were performed using parametric or non-parametric Student’s *t*-test. *p* Values of <0.05 were considered to be significant (*p < 0.05; **p < 0.01; ***p < 0.001; ****p < 0.0001). Correlations were calculated using the Pearson correlation coefficient test. Principal component analysis (PCA) was conducted using Qlucore Omics Explorer v3.0 (Qlucore AB, Lund, Sweden). In the PCA model, *t*-test was used to compare two groups.

### Ethics statement

Written informed consent was obtained in all cases as part of protocols approved by the local ethics committee of the Hannover Medical School. All investigations conformed to the principles espoused in the Declaration of Helsinki.

## Results

### CD8^+^ T cells from patients with CHC show elevated co-regulatory receptor expression

**Figure 1:**
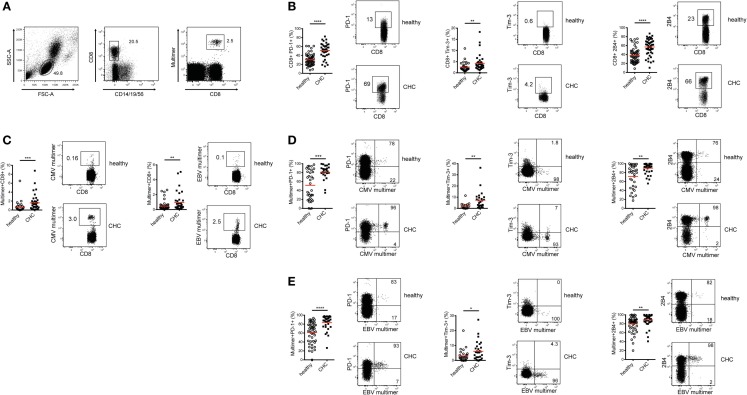
**Expression of co-regulatory receptors on total and CMV/EBV-specific CD8^+^ T cells in CHC patients and healthy controls *ex vivo***. **(A)** Exemplary FACS staining showing the gating strategy used to identify total and MHC-I multimer-positive virus-specific CD8^+^ T cells. **(B)** Expression of PD-1, Tim-3, and 2B4 is elevated on total CD8^+^ T cells in patients with CHC (*n* = 36 for PD-1 and Tim-3, *n* = 46 for 2B4) compared to healthy individuals (*n* = 54 for PD-1 and Tim-3, *n* = 31 for Tim-3). **(C)** Frequencies of CMV- and EBV-specific CD8^+^ T cells are elevated in patients with CHC (*n* = 36 for CMV and *n* = 38 for EBV) compared to healthy individuals (*n* = 34 for CMV and *n* = 49 for EBV). Expression of PD-1, Tim-3, and 2B4 is elevated on **(D)** CMV-specific (Healthy:CHC, PD-1 *n* = 34:25, Tim-3 *n* = 16:25, 2B4 *n* = 34:36) and **(E)** EBV-specific CD8^+^ T cells (Healthy:CHC, PD-1 *n* = 32:30, Tim-3 *n* = 29:30, 2B4 *n* = 49:38) in these CHC patients compared to the healthy blood donors. Red horizontal bars indicate mean values. Representative FACS plots are shown. Cells were gated on lymphocytes and total CD8^+^ T cells after exclusion of dump channel.

In a previous report, we demonstrated that the co-regulatory receptor 2B4 was increased in expression in patients with CHC compared to healthy individuals (20). To extend this further and investigate how a broader array of co-regulatory receptor expressions are influenced by a persistent HCV infection, frequencies of total CD8^+^ T cells expressing PD-1, Tim-3, and 2B4 were assessed in patients with CHC and healthy controls *ex vivo* (Figures 1A,B). Patients with CHC displayed significantly higher frequencies of PD-1-, Tim-3-, and 2B4-expressing CD8^+^ T cells compared to healthy controls (Figure 1B). While the frequency of PD-1-expressing CD8^+^ T cells was 1.70-fold higher in patients with CHC, that of Tim-3 and 2B4 were 1.65 and 1.49-fold, respectively, higher as compared to healthy donors.

To specifically evaluate the expression patterns of PD-1, Tim-3, and 2B4 on antigen-specific CD8^+^ T cells in both cohorts, we focused on CMV and EBV; two common human pathogens with large CD8^+^ T cell memory pools that are easily detectable *ex vivo*. Patients with CHC harbored significantly higher frequencies of CMV- and EBV-specific CD8^+^ T cells *ex vivo* in relation to healthy individuals (Figure 1C). In fact, we evaluated the cohort CHC only as those from the healthy blood donors were unavailable for regulatory reasons. We observed that the frequency of CMV-, but not EBV-specific CD8^+^ T cells *ex vivo* positively correlated with age (data not shown). When the direct *ex vivo* expression of co-regulatory receptor molecules on CMV-specific CD8^+^ T cells was compared between patients with CHC and healthy individuals, we observed that the frequencies of PD-1-, Tim-3-, and 2B4-expressing CMV-specific CD8^+^ T cells were significantly increased in the latter (Figure 1D). A similar observation was made for EBV-specific CD8^+^ T cells (Figure 1E). Unsurprisingly, the simultaneous co-expression of all three co-regulatory receptors was similarly up-regulated on CMV- and EBV-specific CD8^+^ T cells in CHC patients compared to healthy individuals (data not shown). To further investigate whether age is associated with co-regulatory receptor expression, we used data from patients with CHC. Here, no correlation was observed between co-regulatory receptor expression and age. Patients with CHC were further categorized into two groups: young (<50 years) and old (>50 years). We still did not observe any differences in co-regulatory receptor expression between the two groups (data not shown). In summary, persistent infection with HCV was associated with an altered phenotype of both bulk and CMV- and EBV-specific CD8^+^ T cells *ex vivo* with a shift toward co-regulatory receptor upregulation.

### *In vitro* peptide stimulation pronounces the differential co-regulatory receptor expression on CMV-/EBV-specific CD8^+^ T cells in patients with CHC

**Figure 2:**
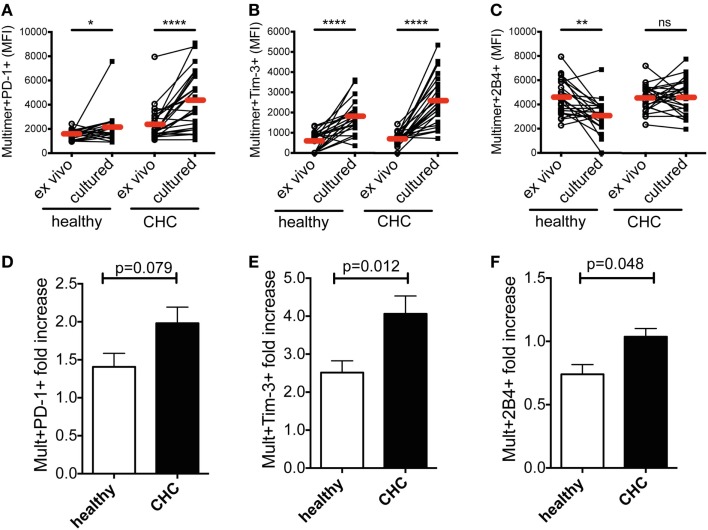
**Effect of *in vitro* peptide stimulation on co-regulatory receptor expression by CMV-/EBV-specific CD8^+^ T cells in patients with CHC and healthy individuals**. A comparison of the mean fluorescence intensities (MFI) of **(A)** PD-1, **(B)** Tim-3, and **(C)** 2B4 expressions on CMV-/EBV-specific CD8^+^ T cells in healthy individuals (*n* = 12 for CMV; *n* = 10 for EBV) and patients with CHC (*n* = 12 for CMV, *n* = 14 for EBV) revealed a stronger increase of expression in CHC patients upon peptide stimulation *in vitro*. Indicated are individual values of co-regulatory receptor expression *ex vivo* and upon culture *in vitro*. Red horizontal bars indicate mean values. The increase of expression was also revealed by comparing the fold increase of expression of **(D)** PD-1, **(E)** Tim-3, and **(F)** 2B4 upon *in vitro* peptide stimulation in relation to the *ex vivo* expression. *p*-Values are indicated. Cells were gated on lymphocytes and total CD8^+^ T cells after exclusion of dump channel.

Next, we investigated the dynamics of co-regulatory receptor expression on CMV- and EBV-specific CD8^+^ T cells from the two cohorts upon peptide stimulation. PBMCs from randomly selected patients of either cohort were stimulated with virus-specific peptides *in vitro* for 10 days and subsequently stained with MHC class I multimers. The expression of PD-1, Tim-3, and 2B4 was investigated. Overall, CMV- and EBV-specific CD8^+^ T cells showed a similar expression pattern with no significant differences between them as demonstrated in Figure 1. We therefore performed all subsequent analyses with the two cell populations together. Evaluating the two virus-specific CD8^+^ T cell populations together, we observed that the expression of PD-1 and Tim-3 was particularly up-regulated upon peptide stimulation *in vitro* (Figures 2A,B and 2D,E; Figure S1 in Supplementary Material). This pattern of response was observed irrespective of the sample source; CHC patients or healthy blood donors. However, the comparative potentiation of PD-1 and Tim-3 expressions was stronger for CMV- and EBV-specific CD8^+^ T cells in CHC patients than in healthy individuals (Figures 2A,B and 2D,E). This was evident in the superior fold increases in the CHC patients (PD-1 = 1.82-fold, Tim-3 = 3.72-fold) compared to the healthy controls (PD-1 = 1.36-fold, Tim-3 = 3.16-fold). The expression of 2B4 was down-regulated but this was only noticeable on CMV- and EBV-specific CD8^+^ T cells in healthy individuals (fold decrease = 0.68) compared to patients with CHC where they remained fairly stable (fold increase = 1.01; Figures 2C,F; Figure S1 in Supplementary Material). These data indicate that the inflammatory milieu in CHC patients may precondition CMV- and EBV-specific CD8^+^ T cells to up-regulate co-regulatory receptor expression.

### CMV-/EBV-specific CD8^+^ T cells from CHC patients display an elevated proliferation and cytokine production

**Figure 3:**
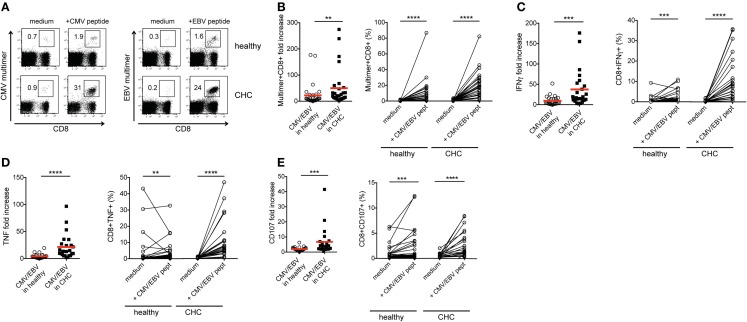
**Proliferation and cytokine production *in vitro* by CMV-/EBV-specific CD8^+^ T cells from CHC patients and healthy individuals**. PBMCs from patients with CHC (*n* = 32) and healthy individuals (*n* = 28) were stimulated with CMV- or EBV-specific peptides *in vitro* for 10 days. **(A)** Representative FACS plots for staining of CMV- and EBV-specific CD8^+^ T cells and **(B)** pooled data show an increased fold expansion of CMV-(Healthy: CHC, *n* = 16:13) and EBV-(Healthy:CHC, *n* = 16:15) specific CD8^+^ T cells in CHC patients compared to healthy individuals. **(C)** Fold increases of IFNγ- and **(D)** TNF production as well as **(E)** CD107-expression on CMV-(Healthy:CHC, *n* = 14:11) and EBV-(Healthy:CHC, *n* = 13:13) specific CD8^+^ T cells upon stimulation with CMV- or EBV-specific peptides *in vitro* were stronger in CHC patients as compared to healthy controls. Graphs on the left hand side show fold increases induced by peptide stimulation in comparison to unstimulated (medium only) controls. Right hand graphs show individual absolute frequencies for healthy individuals and CHC patients found for medium controls and peptide-stimulated cells. Red horizontal bars indicate mean values. Cells were gated on lymphocytes and total CD8^+^ T cells after exclusion of dump channel.

In the light of the dichotomy between the dual roles of co-regulatory receptor expression in T cell exhaustion and activation, we next assessed the proliferative, cytokine-producing, and cytotoxic capacities of peptide-stimulated CMV- and EBV-specific CD8^+^ T cells from the two cohorts. The proliferation of CMV- and EBV-specific CD8^+^ T cells was significantly enhanced in patients with CHC compared to healthy individuals (Figures 3A,B; Figures S2A and S3A in Supplementary Material). This was evident in the 2.23-fold higher expansion observed in CHC patients (mean fold increase = 49.78) in relation to that of healthy individuals (mean fold increase = 22.35). Similarly, the mean fold increases of IFNγ and TNF production as well as CD107a/b expression (a surrogate marker for cytotoxicity) were all stronger in patients with CHC (37.54, 21.36, and 35.81-fold, respectively) compared to healthy controls (9.49, 4.16, and 2.02-fold, respectively) (Figures 3C–E; Figures S2B–D, S3B–D, and S4 in Supplementary Material). This suggests that the stronger induction of co-regulatory receptor expression by CMV- and EBV-specific CD8^+^ T cells upon *in vitro* peptide stimulation may culminate in an enhanced proliferation and functional responses rather than attenuating them.

This observation of an enhanced co-regulatory receptor expression on CD8^+^ T cells and their nevertheless strong functional capacity is contrary to the often described and accepted inhibitory effect of PD-1 and Tim-3 and partially also 2B4. Indeed, to show that the co-regulatory receptor-positive cells might nevertheless effectively proliferate, we grouped the samples of healthy and CHC individuals according to their *ex vivo* frequencies of expression of PD-1, Tim-3, and 2B4 into high and low groups. Here, all these groups showed a similar proliferation of CMV- and EBV-specific CD8^+^ T cells (data not shown), no differences in proliferative responses in correlation to the expression of these co-regulatory receptors were apparent in healthy individuals or CHC patients.

### Plasma cytokine patterns differ between CHC patients and healthy individuals and correlate with expression of co-regulatory receptor expression

**Figure 4:**
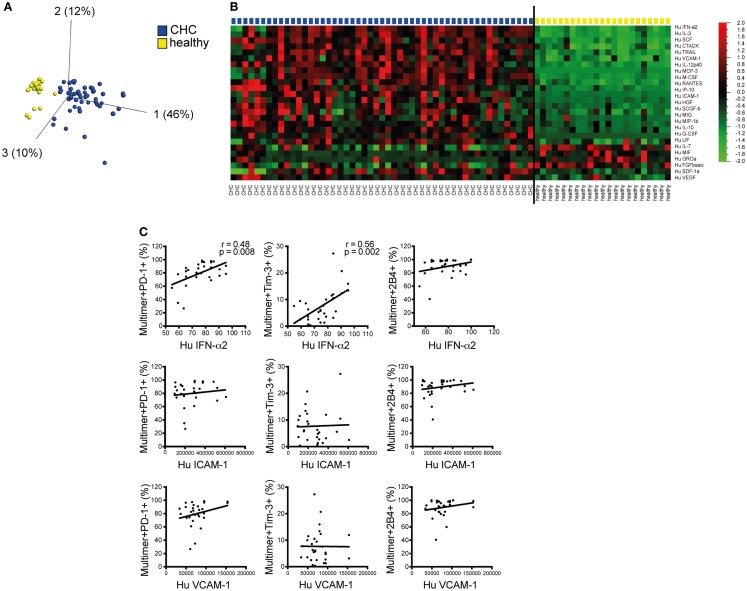
**Differences of and association of plasma cytokine/chemokine levels with *ex vivo* expression of distinct co-regulatory receptors on CMV/EBV-specific CD8^+^ T cells in CHC patients**. Fifty cytokines and chemokines were measured in the plasma of CHC patients without prior treatment and healthy controls by a multiplex assay. **(A)** Principal component analysis showed a distinct clusterization of CHC patients (blue) and healthy individuals (yellow) based on the differential concentrations of plasma cytokines and chemokines. **(B)** Heat map comparing plasma cytokine/chemokine levels showed cytokines and chemokines with significantly differential concentrations between CHC patients and healthy individuals. **(C)** Correlation analysis of IFN-α2, VCAM-1 (CD106), and ICAM-1 (CD54) with co-regulatory receptor expression revealed a positive correlation of IFN-α2 plasma levels with PD-1 and Tim-3 expression *ex vivo*. Each patient’s FACS data were correlated individually to the same patient’s cytokine data obtained from the multiplex assay. *p*- and *r*^2^-values are indicated. Cells were gated on lymphocytes and total CD8^+^ T cells after exclusion of dump channel.

The foregoing findings prompted us to investigate the possible mechanism behind the altered phenotype and function of CD8^+^ T cells in persistent HCV infection as observed. As cytokines and chemokines produced during chronic inflammation in patients with CHC may substantially influence immune cells, we aimed to investigate their possible contribution. Thus, we quantified several cytokines and chemokines (*n* = 50) in the plasma of patients with CHC and healthy individuals using a multiplex bead assay. Using PCA, the differential concentrations of different cytokines and chemokines in CHC patients and healthy individuals resulted in a distinct clusterization of members of each cohort (Figure 4A). Particularly, inflammatory mediators and proteins, such as IFNα2, IL-3, SCF, IL-2Rα, CTACK (CCL27), TRAIL (CD253), VCAM-1 (CD106), IL-12p40, MCP-3 (CCL7), M-CSF, RANTES (CCR5), IP-10 (CXCL10), ICAM-1 (CD54), HGF, SCGF-β, MIG (CXCR3), MIP-1β (CCL4), IL-18, G-CSF, LIF, SDF-1α (CXCL12), and VEGF, were all significantly elevated in patients with CHC as compared to healthy individuals (Figure 4B). Other mediators including IL-17, MIF, GROα, and FGF were, however, down-regulated in patients with CHC. To identify a possible link to the phenotype of CD8^+^ T cells, we then performed correlation analyses between the levels of plasma cytokines and chemokines and co-regulatory receptor expression on CMV- and EBV-specific CD8^+^ T cells *ex vivo* on an individual basis comparing the respective patient’s FACS data with the cytokine values obtained from the multiplex assay (Figure 4C). For representation purposes, we selected the three recombinant human proteins VCAM-1, ICAM-1, and IFNα-2a that were further used for *in vitro* stimulation experiments. These proteins were selected based on their elevated plasma levels in CHC patients, literature-proven T cell co-stimulatory effects inducing cell signaling, presence of their respective receptors on T cells as well as their correlation with PD-1, Tim-3, or 2B4 *ex vivo* expression. Our results demonstrated a significant positive correlation of IFNα-2a plasma levels with PD-1 and Tim-3 expressions (Figure 4C). The seemingly positive correlation between IFNα-2a and 2B4 expressions, however, did not reach statistical significance. Apart from IFNα-2a other components of the HCV-induced inflammatory milieu such as IP-10, SDF, RANTES, SCF, and IL-18 positively correlated with at least one of PD-1, Tim-3, or 2B4 expression *ex vivo* (data not shown). By contrast, no correlation was observed between plasma concentrations of VCAM-1 and ICAM-1 (two adhesion molecules that were also up-regulated in CHC patients) with PD-1, Tim-3, or 2B4 expression (Figure 4C). These results hinted a role of IFNα-2a in influencing co-regulatory receptor upregulation on CMV- and EBV-specific CD8^+^ T cells in patients with CHC.

### *In vitro* IFNα stimulation converted the phenotype of CMV-/EBV-specific CD8^+^ T cells in healthy individuals to those in patients with CHC

**Figure 5:**
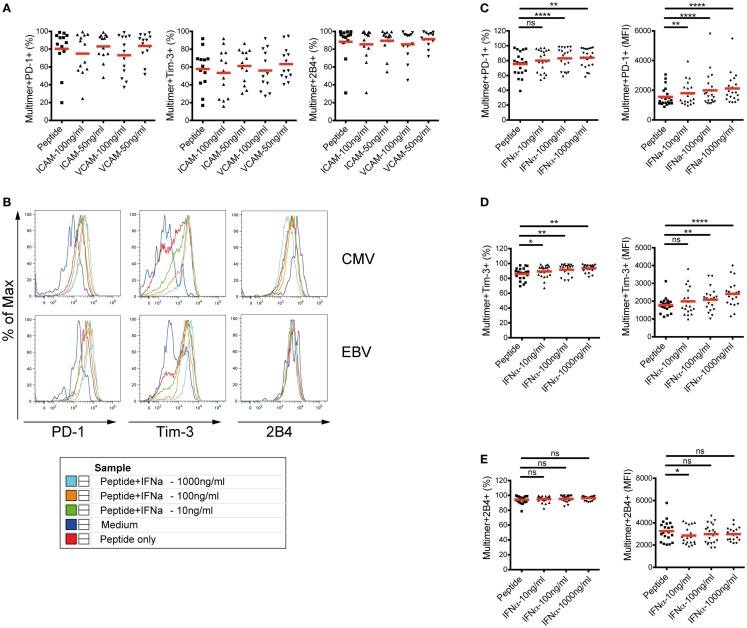
**Effect of *in vitro* cytokine/chemokine stimulation on the phenotype of CMV- and EBV-specific CD8^+^ T cells in healthy blood donors**. Expression of PD-1, Tim-3, and 2B4 was analyzed on CMV- and EBV-specific CD8^+^ T cells in PBMC from healthy blood donors (*n* = 16) after stimulation with selected proteins together with virus-specific peptides. **(A)** Stimulation with different concentrations of VCAM-1 (CD106) or ICAM-1 (CD54) did not alter the expression of PD-1, Tim-3, or 2B4 on CMV-/EBV-specific CD8^+^ T cell responses (*n* = 13). **(B)** Representative FACS histogram overlays showing the frequencies and the expression intensities (MFIs) of PD-1, Tim-3, and 2B4 upon stimulation with different concentrations of IFN-α2. One healthy individual is shown as representative. Stimulation with IFNα-2 *in vitro* resulted in a consistent upregulation of both the frequency (left) and MFI (right) of **(C)** PD-1, and **(D)** Tim-3 expression or **(E)** down-regulation of 2B4 expression on CMV-/EBV-specific CD8^+^ T cells in a dose-dependent manner. *In vitro* stimulation assay with VCAM-1 and ICAM-1 was performed in a separate experiment from that with IFNα-2. Red horizontal bars indicate mean values. Cells were gated on lymphocytes and total CD8^+^ T cells after exclusion of dump channel.

To further investigate whether the alterations of PD-1, Tim-3, and 2B4 expression patterns are indeed induced by cytokine/chemokine stimulations, we stimulated PBMC *in vitro* from healthy blood donors with different concentrations of selected recombinant human proteins for 10 days in the presence of CMV- or EBV-specific peptides. Our selected recombinant human proteins included VCAM-1, ICAM-1, and IFNα-2a. Here, our aim was to assess the ability of the selected soluble mediators to convert the phenotype of CMV- and EBV-specific CD8^+^ T cells from healthy donors into those that were found in patients with CHC. In our analyses, no effect of VCAM-1 or ICAM-1 stimulation on the expression of PD-1, Tim-3, or 2B4 on CMV- and EBV-specific CD8^+^ T cells was evident (Figure 5A). Interestingly, however, stimulation with IFNα-2a *in vitro* resulted in a consistent dose-dependent upregulation of PD-1 and Tim-3 expressions. This was reflected not only in the frequencies of positive cells but also in the expression levels (MFI) (Figures 5B–D). The expression of 2B4 on IFNα-stimulated CMV- and EBV-specific CD8^+^ T cells in healthy individuals was reminiscent of the phenotype we observed earlier in CHC patients (Figure 5E). Although Tim-3 expression was marginally up-regulated, stimulation of PBMCs from healthy individuals with only IFNα-2a without any virus-specific peptides, however, did not result in any considerable effect suggesting an antigen-dependent effect of IFNα-2a stimulation on the modulation of T cell co-regulatory receptor expression (data not shown).

### Treatment-induced HCV clearance resulted in a partial reversion of the phenotype of CMV-/EBV-specific CD8^+^ T cells

**Figure 6:**
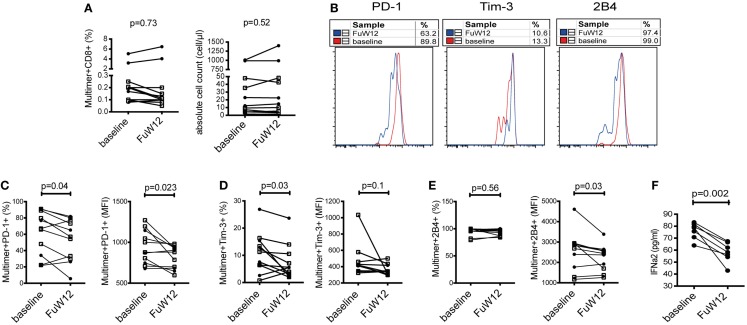
**Phenotypic characterization of co-regulatory receptor expression by CMV-/EBV-specific CD8^+^ T cells *ex vivo* in CHC patients clearing the infection upon antiviral therapy**. PBMCs from seven patients who had cleared HCV infection after receiving IFN-free antiviral therapy with sofosbuvir were analyzed at baseline (BL) and 12 weeks after end of therapy (FuW12). **(A)** Absolute frequencies (left) and absolute cell numbers (right) of CMV-/EBV-specific CD8^+^ T cells *ex vivo* did not significantly differ between baseline and FuW12. **(B)** Representative FACS histogram overlays showing staining of PD-1, Tim-3, and 2B4 expressions on EBV-specific CD8^+^ T cells *ex vivo* showing a partial decrease of expression at FuW12 (blue line) as compared to baseline (red line). A loss of the frequencies (left) and mean fluorescence intensity (right) of **(C)** PD-1, **(D)** Tim-3, and **(E)** 2B4 expressions on CMV-/EBV-specific CD8^+^ T cells was visible at FuW12 indicating a loss and thus partial reversion of their phenotype upon antiviral treatment for HCV. **(F)** Plasma IFN-α2 levels at baseline and FuW12 were analyzed by multiplex assay revealing a significant reduction of IFN-α2 levels upon therapy-induced clearance of HCV. Cells were gated on lymphocytes and total CD8^+^ T cells after exclusion of dump channel.

Our previous data demonstrated that the cytokine milieu can impact the phenotype of CMV- and EBV-specific CD8^+^ T cells leading to an enhanced expression of co-regulatory molecules. We next asked whether an antiviral therapy-induced clearance of HCV RNA would then lead to a reversion of the phenotype of CMV- and EBV-specific CD8^+^ T cells to that found in healthy individuals by causing a decrease in PD-1, Tim-3, and 2B4 expressions. To address this, we used PBMC from seven patients with CHC who had undergone successful antiviral treatment based on the novel IFN-free sofosbuvir administration. CMV- and EBV-specific CD8^+^ T cells from these samples were analyzed *ex vivo* at baseline (before treatment) and follow-up week 12 (FuW12). We did not observe any differences in the frequency of CMV-/EBV-specific CD8^+^ T cells between baseline and FuW12 *ex vivo* and also not in the absolute cell count of these cells (Figure 6A). This observation was the same for both CMV- and EBV-specific CD8^+^ T cells, demonstrating that the survival of these cells is not affected by the treatment. However, comparing the co-regulatory receptor expression between the two time points, we could observe a significant reduction in both the frequency (%) and MFI of PD-1 expression at FuW12 (Figures 6B,C). The frequency of Tim-3-expressing CMV-/EBV-specific CD8^+^ T cells also decreased significantly at FuW12, although this was not reflected in the MFI (Figure 6D). The expression of 2B4 remained almost unaltered between baseline and FuW12 except for a borderline significance, which was recorded for its MFI (Figure 6E). In accordance with this, the plasma levels of cytokines/chemokines measured by multiplex assay changed upon therapy-induced resolution of infection (data not shown). Importantly, IFNα levels decreased significantly until FuW12 (Figure 6F), which is in correlation to the observed change of the phenotype of CMV- and EBV-specific CD8^+^ T cells. Overall, this data further strengthen the hypothesis that the phenotype of CMV- and EBV-specific CD8 T^+^ cells is affected by the inflammatory cytokine milieu present during persistent HCV infection.

## Discussion

Overexpression of distinct co-regulatory receptors such as PD-1, CTLA-4, Tim-3, Lag-3, and 2B4 has been previously described and strongly considered as a fingerprint for exhausted T cells. Particularly, in many persistent virus infections where T cells have been shown to have an impaired proliferative capacity and functionality, upregulation of various co-regulatory receptors have been observed. In this context, it has been suggested that co-regulatory receptor overexpression is a way the immune system prevents uncontrolled inflammation and pathology due to the constant antigen stimulation. However, antigen stimulation alone does not seem to be the only driving force behind co-regulatory receptor upregulation. Indeed, several studies have demonstrated both antigen-dependent and -independent mechanisms that are capable of increasing co-regulatory receptor expression. For example, antigen specificity, cell differentiation, and the microenvironment were found to strongly influence the expression of co-regulatory receptors on CD8^+^ T cells (21–23). Using HCV as a model of persistent virus infection in humans, we comprehensively investigated the influence inflammatory mediators induced during persistent infections have on co-regulatory receptor expression by CD8^+^ T cells specific for the common pathogens CMV and EBV. Both CMV and EBV are particularly ubiquitous in the human population where they usually have a lifelong persistence in the infected host. Virus-specific CD8^+^ T cell populations targeting the two viruses are often larger and *ex vivo* detectability is high. These attributes informed our choice of studying the two viruses and afforded us the opportunity to examine the effects of HCV’s persistence on an unrelated antigen-specific T cell population in clear, unambiguous terms using the MHC-I multimer technology.

Evaluating co-regulatory receptor expression by antigen-specific CD8^+^ T cells, we observed that PD-1-, Tim-3-, and 2B4-expressing CMV- and EBV-specific CD8^+^ T cells were enriched in patients with CHC compared to their counterparts in uninfected controls. The increased frequencies of CMV-specific CD8^+^ T cells expressing Tim-3 in patients with CHC compared to uninfected controls was previously shown (24). Our findings here, derived from a larger group of patients, thus confirm and significantly extend the previous report even further with two additional co-regulatory molecules (PD-1 and 2B4) prominent in persistent infections. We further observed that persistent HCV infection not only influenced co-regulatory receptor expression by antigen-specific CD8^+^ T cells of viruses unrelated to HCV but also impacted the phenotype of total CD8^+^ T cells. The strikingly high expression of PD-1, Tim-3, and 2B4 by the total CD8^+^ T cell population in persistent HCV infection is indicative of the pervasive global influence persistent HCV has on the phenotype of T cells, beyond those that are HCV-specific. We further have data published recently showing that the enhanced functionality of immune cells during CHC is not restricted to CD8^+^ T cells, but that a similar elevated functionality can be observed for NK cells as well (25). Certainly, these observations called into question the actual role of co-regulatory receptor expression on antiviral T cell immunity in disease. Indeed, the association of functional exhaustion to the elevated expression of co-regulatory receptors in patients with CHC has been well established (3, 26). However, apart from this report, high PD-1 and 2B4 expressions on functionally competent CMV- and/or EBV-specific CD8^+^ T cells in patients with CHC have also been demonstrated before (16, 20, 27). Moreover, we demonstrated in a recent report that PD-1 and 2B4 expressions by CMV- and EBV-specific CD8^+^ T cells in patients with CHC were significantly higher compared to exhausted HCV-specific CD8^+^ T cells in the same patients (28). Again, Kroy et al. who extensively characterized co-regulatory receptor expression on CD8^+^ T cells on IHLs also revealed that IHLs from liver tissue without infection, inflammation, or malignancy displayed the same patterns of PD-1 and 2B4 expressions as those with HCV infection (22). Strengthened by previous observations, our findings here therefore water down the heightened assumption and reference of co-regulatory receptor molecules as “classical markers of exhaustion.” Further, it strongly supports the notion that enrichment of co-regulatory receptor expression (especially PD-1 and 2B4) may be a mere physiological response to the microenvironment and not necessarily a reflection of a failing immune response in disease.

In many persistent virus infections, the expression of multiple co-regulatory receptors has been tightly linked to a reduced proliferation and cytokine production (29, 30). In recent years, however, it is becoming increasingly evident that co-regulatory receptor expression may not solely drive the functional impairment of effector CD8^+^ T cells. Many independent investigations have otherwise shown that co-regulatory receptor-positive CD8^+^ T cells in peripheral blood of healthy donors are not necessarily functionally impaired (23, 31, 32). Instead, PD-1 expression correlated with positive T cell function and with activation markers such as CD38 in HIV infection as reviewed in Ref. (33) or CD137 in breast cancer studies (34). Guided by these observations, we sought to ascertain the functional consequence of the HCV-induced upregulation of PD-1, Tim-3, and 2B4 on the CMV- and EBV-specific CD8^+^ T cells in CHC patients compared to healthy donors. We observed significantly enhanced functional responses upon peptide stimulation, which was distinctive to CMV- and EBV-specific CD8^+^ T cells exposed to the inflammatory milieu in persistent HCV infection. Ostensibly, this observation was made concomitant to a relative upregulation of co-regulatory receptor expression through TCR stimulation with cognate peptides *in vitro*. This observation was exceptionally interesting as it particularly linked immune activation with co-regulatory receptor upregulation and improved functional responses (at least for CMV- and EBV-specific CD8^+^ T cells) as has been suggested before (23). Further, stratifying our samples by the *ex vivo* expression levels of co-regulatory receptors revealed that there is no difference in the *in vitro* proliferative responses between *ex vivo* low and high expressing samples and this held true for both healthy individuals and CHC patients. Overall, these observations suggest that PD-1 or Tim-3 expression *per se* do not necessarily make T cells exhausted and dysfunctional, but that additional and so far unknown events appear to be crucial to convert these molecules into actual inhibitors. Further, these data point to a much profound conditioning of CMV- and EBV-specific CD8^+^ T cells by the inflammatory milieu in patients with CHC, which alters co-regulatory receptor expression, and the cells benefiting thereof with an improved functional responses.

The changes in phenotype and function during CHC were visible and common for both CMV- and EBV-specific CD8^+^ T cells with no major differences apparent. This suggests that broad and general factors seem to drive these changes and not virus-specific ones. This becomes especially important in the light of a recent publication showing that CMV infection is one of the major non-heritable factors impacting the immune phenotype and response in humans (35). Despite that we can observe a slight correlation of the frequencies of CMV-specific CD8^+^ T cells with age in our cohort of CHC patients, which is absent for EBV-specific CD8^+^ T cells, again in both cases the change of frequency, phenotype, and function can be seen for both virus specificities. Further, also among our healthy cohort individuals are included that are infected with CMV as well, thus a CMV-specific impact as described by Brodin et al. (35) does not seem to be the major force driving our observations. However, our findings are nevertheless in line with the publication by Brodin et al., as we also show that environment influences and other infections (in our case HCV) seem to alter and shape the immune system.

The presence of assortments of cytokine receptors on effector and memory antiviral CD8^+^ T cells enable them to respond to shifts in the cytokine milieu and may even allow them to circumvent their requirements for antigen-dependent activation (36). Recently, effector and memory CD8^+^ T cells’ sensitivity to an expansive range of cytokines has been profiled (37). The effects of inflammatory mediators on the phenotypic changes of CMV- and EBV-specific CD8^+^ T cells observed in this study was therefore not so surprising following these previous reports. Interestingly, several of the inflammatory cytokines and plasma proteins from our study here, such as ICAM-1, VCAM-1, IFN-α2, SDF-1α, and IL-18, that showed elevated concentrations in patients with CHC compared to healthy individuals have been associated with T cell activation (38–40). In fact, we show in this study for the first time that IFN-α2 was not only the differentially dominant component of the HCV-induced inflammatory mediators but also positively correlated with co-regulatory receptor expression *ex vivo*. Interestingly, IFN-α2 further caused an expected upregulation of PD-1 and Tim-3 expression on CMV- and EBV-specific CD8^+^ T cells in healthy individuals *in vitro*. However, other mediators such as the adhesion molecules ICAM-1 and VCAM-1 failed to replicate any of these IFN-α2’s *ex vivo* behavior or *in vitro* effects. Nevertheless, these findings further confirm the profound effect some inflammatory mediators such as type I interferon may have on the phenotype of antiviral CD8^+^ T cells. It further suggests a specific importance of IFN-I in regulating antiviral T cell immunity through co-regulatory receptor expression in order to protect them against killing by NK cells as a regulatory immune function as suggested previously (41).

The expression of 2B4 instead showed a decline of expression after INF-α2 stimulation *in vitro*, while the frequency of expression of PD-1 and Tim-3 was consistently increased. This different behavior of 2B4 upon stimulation might be based on the fact that 2B4 is not a classical inhibitory receptor, but it rather has a dual function able to induce at least two different downstream signaling pathways, which subsequently elicit different cellular responses (i.e., either activation or inhibition) (42).

Previous studies by Wang et al. in patients with chronic hepatitis B showed a reduction of PD-1 and Tim-3 expressions on CD8^+^ T cells following antiviral treatment against HBV (43). However, to our knowledge, no study has so far dissected whether the cessation of inflammation in patients with CHC revert the phenotype of CMV- and EBV-specific CD8^+^ T cells hitherto induced by the inflammatory environment. The introduction of the novel IFN-free therapy to cure HCV infection allowed us the unique opportunity to investigate the effects therapy-induced HCV clearance has on co-regulatory receptor expression on CMV- and EBV-specific CD8^+^ T cells. Of note, HCV RNA shows a very rapid decline early after start of IFN-free therapy and the inflammatory activity of liver disease already dramatically reduces 7–14 days after therapy start (44) suggesting that cytokine patterns normalize rather soon during therapy. Comparing co-regulatory receptor expression between baseline and 12 weeks after the end of antiviral therapy, we observed that both the frequency and MFI of PD-1 expression by CMV- and EBV-specific CD8^+^ T cells reduced significantly after viral clearance. While only the frequency of Tim-3-expressing CMV- and EBV-specific CD8^+^ T cells experienced a significant decline, that of 2B4 was reflected only in its MFI. These differential dynamics of PD-1, Tim-3, and 2B4 with an apparently earlier and faster decline of PD-1 expression is noteworthy and it would be interesting to see the long-term effects of successful HCV clearance. Further, this partial normalization of co-regulatory receptor expression by pathogens unrelated to HCV following a treatment that directly targets HCV reflects the development of antigen-independent immune homeostasis upon antiviral therapy in patients with CHC.

The change of the phenotype of CMV- and EBV-specific CD8^+^ T cells in CHC patients is accompanied by an elevated frequency of cells and an enhanced functionality *in vitro*. While the change of phenotype might at least partially be explained by the influence of IFNα, which is drastically elevated during CHC, the means behind the increased functionality remains unclear. One possible explanation might be a shift in the expression of STAT transcription factors and subsequently induced ISGs, as we and others could previously show to be the case for the enhanced functionality of NK cells in CHC patients (25, 45). It might well be that similar mechanisms also apply for CD8^+^ T cells as demonstrated recently in mice (46). However, preliminary data suggest that mechanism(s) other than IFNα stimulation seems to drive these changes. Despite a clear decline of plasma IFNα levels associated with a reversion of the phenotype of CMV- and EBV-specific CD8^+^ T cells in CHC patients successfully treated with the direct-acting antivirals sofosbuvir as shown here, the frequency and functionality of these cells did not change at FuW12 (data not shown).

The data presented here show the broad and pervasive influence HCV has on the immune system and other immune cells, besides the HCV-specific ones. Here, this manifests itself by the alteration of the properties of CMV- and EBV-specific CD8^+^ T cells and by the change of plasma cytokines in patients with CHC. It is justified to assume that these effects also extend to other cells of the adaptive (i.e., besides CMV- and EBV-specific CD8^+^ T cells) and innate immune system (e.g., NK cells, dendritic cells, monocytes). Apart from the hitherto described impact of IFNα, several other reasons and mechanisms might contribute as well. For instance, other cytokines and inflammatory mediators elevated or decreased during persistent infection might contribute to the conditioning of immune cells. In this regard, also the homeostatic proliferation of immune cells regulated by several cytokines might be affected leading to the here described alterations. Further, direct antigen-specific effects generated, e.g., by T cell cross-reactivity might apply as described for other scenarios (47, 48). Importantly, our data presented here are in line and extend the influence that viral infections might have on immune cell populations, phenotype, and function as recently described for CMV in studies of twin pairs (35) and enlarge the portfolio of influencing factors to include HCV as well.

In summary, this study presents a two-fold level of relevance. First, they indicate that a persistent infection with HCV cannot be considered in isolation as it may have far-ranging effects on the global CD8^+^ T cell population as well as on antigen-specific CD8^+^ T cell immunity against unrelated pathogens such as CMV and EBV. Here, persistent HCV-induced up-regulated expression of co-regulatory receptors PD-1, Tim-3, and 2B4 on *in vitro*-verified, functionally competent CMV- and EBV-specific CD8^+^ T cells. This mechanism suggests that distinct co-regulatory receptors are expressed concomitantly with various inflammatory factors, which boosts functionality but negatively regulate the immune response upon reaching a threshold. Second, they provide a handle to investigate the mechanisms regulating epitope-specific CD8^+^ T cell function in general, which might be of clinical relevance considering the rather high incidence of a broad array of co-morbidities observed in patients with CHC. These data further give insight into the regulation of antiviral pathogen-specific CD8^+^ T cell responses, especially during clinically relevant virus co-infections in humans such as HCV/HBV, HCV/HIV, and HBV/HDV. Given the severity of disease associated with co-infections and the difficulty in treating same, such knowledge would particularly be relevant for the development of better therapeutic options.

## Conflict of Interest Statement

The authors declare the following conflicts of interest: Michael Peter Manns, Markus Cornberg, and Heiner Wedemeyer have received honoraria for consulting, speaking, and research support from Abbott Laboratories, Achillion Pharmaceuticals, AbbVie, Bristol Myer Squibb, Gilead Sciences, Janssen Pharmaceutica, Merck Serono KGaA, Novartis, and Roche Pharma AG. The remaining others have no conflict of interest to declare.

## Supplementary Material

The Supplementary Material for this article can be found online at http://journal.frontiersin.org/article/10.3389/fimmu.2015.00270/abstract
