# Supplementary material for: Type I Interferon Elevates Co-Regulatory Receptor Expression on CMV- and EBV-Specific CD8 T Cells in Chronic Hepatitis C
Source: Front Immunol. 2015 Jun 10;6:270. doi: 10.3389/fimmu.2015.00270 (PMC4462106; doi:10.3389/fimmu.2015.00270)
Supplement: Supplementary file 1 [file data_sheet_1.docx]

***Supplementary Material***

**Type I Interferon Elevates Co-regulatory Receptor Expression on CMV- and EBV-specific CD8 T cells in Chronic Hepatitis C**

Solomon Owusu Sekyere^1^, Pothakamuri Venkata Suneetha^1^, Svenja Hardtke^1^, Christine S. Falk ^2^, Julia Hengst^1^, Michael Peter Manns^1,2^, Markus Cornberg^1,2§^, Heiner Wedemeyer^1,2§^, Verena Schlaphoff^1*§^

^1^Department of Gastroenterology, Hepatology and Endocrinology, Hannover Medical School, Hannover, Germany;

^2^Transplantation Immunology; IFB-Tx, Hannover Medical School, Hannover, Germany

^§^Authors contributed equally

*Corresponding Author

## Supplementary Figures

Supplementary Figure 1. Effect of *in vitro* peptide stimulation on co-regulatory receptor expression by CMV-/EBV-specific CD8+ T cells in patients with CHC and healthy individuals. Representative FACS histogram overlays comparing the expression of (A) PD-1, (B) Tim-3 and (C) 2B4 on CMV- (left) and EBV-specific (right) CD8+ T cells of healthy individuals (upper row) and CHC patients (lower row). Blue lines show frequencies and expression intensity (MFI) *ex vivo* and red lines the same after *in vitro* peptide stimulation. Cells were gated on lymphocytes and total CD8+ T cells after exclusion of dump channel.

Supplementary Figure 2. Individual frequencies of the proliferation and cytokine production *in vitro*. PBMCs from healthy individuals and CHC patients were analyzed after *in vitro* stimulation with medium as control and upon CMV/EBV-specific peptide stimulation. (A) Proliferation of CMV- and EBV-specific CD8+ T cells as analyzed by MHC multimer staining. Production of (B) IFNγ, (C) TNF and (D) expression of CD107a/b are shown.

Supplementary Figure 3. Fold increase of proliferation and cytokine production of CMV- and EBV-specific CD8+ T cells *in vitro*. PBMCs from healthy individuals and CHC patients were analyzed for after *in vitro* stimulation. Fold increases of responses upon peptide stimulation were calculated referring to medium controls. Responses for CMV- and EBV-specific CD8+ T cells are displayed separately. (A) Fold increases of CMV- and EBV-specific CD8+ T cells as analyzed by MHC multimer staining. Fold increases of the production of (B) IFNγ, (C) TNF and (D) expression of CD107a/b are shown.

Supplementary Figure 4. Functionality of CMV- and EBV-specific CD8+ T cells upon *in vitro* peptide stimulation. Representative FACS pots showing the IFNγ (upper row) and TNF production (middle row) as well as CD107a/b expression (lower row) upon *in vitro* stimulation with medium as control and peptides specific for CMV (left plots) or EBV (right plots). FACS dot plots are shown for one representative healthy individual and one CHC patient. Cells were gated on lymphocytes and total CD8+ T cells after exclusion of dump channel.
