# Supplementary figures and images for: Type I Interferon Elevates Co-Regulatory Receptor Expression on CMV- and EBV-Specific CD8 T Cells in Chronic Hepatitis C
Source: Front Immunol. 2015 Jun 10;6:270. doi: 10.3389/fimmu.2015.00270 (PMC4462106; doi:10.3389/fimmu.2015.00270)

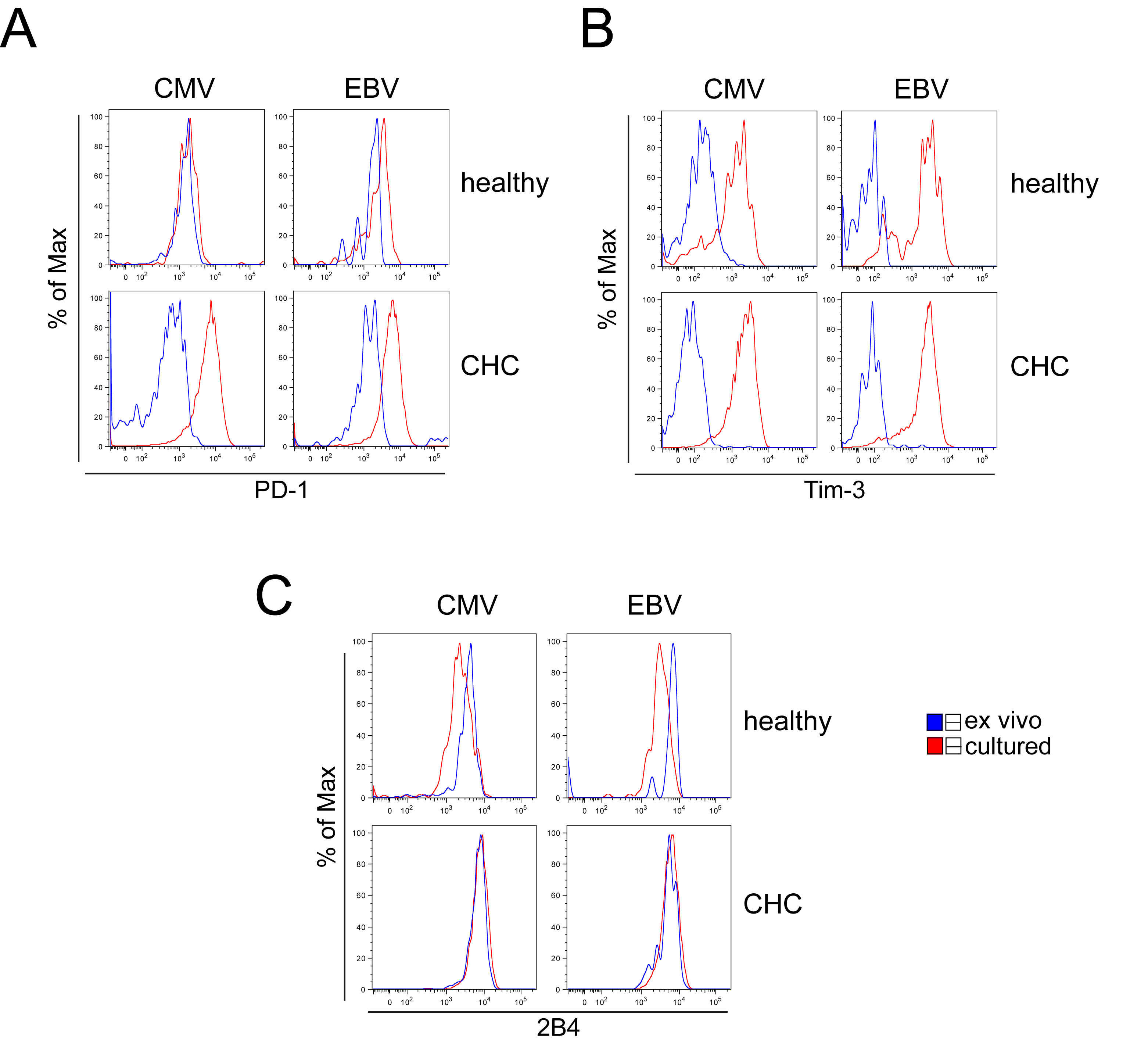

Supplement: Supplementary file 2 [file image_1.tif]

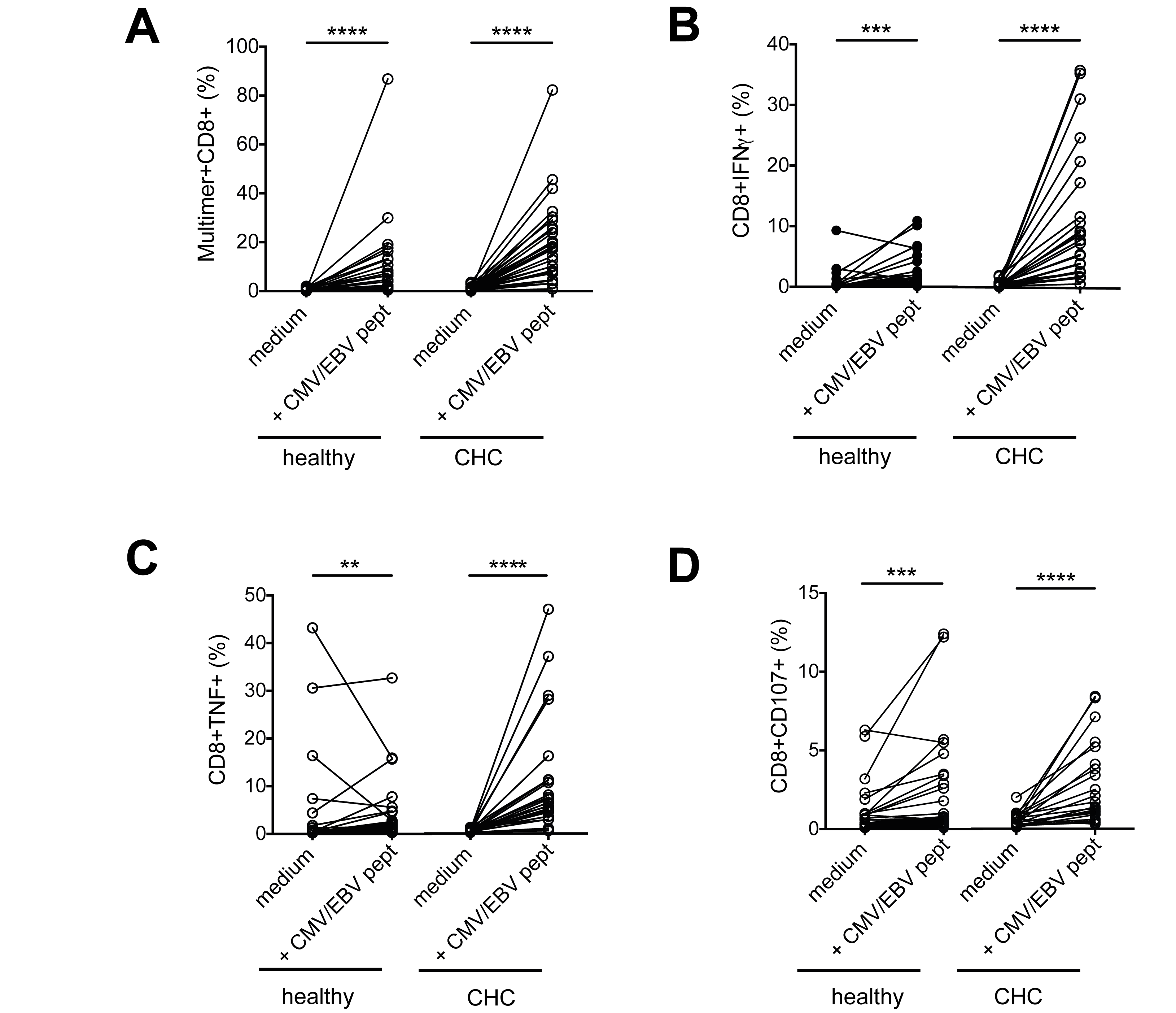

Supplement: Supplementary file 3 [file image_2.tif]

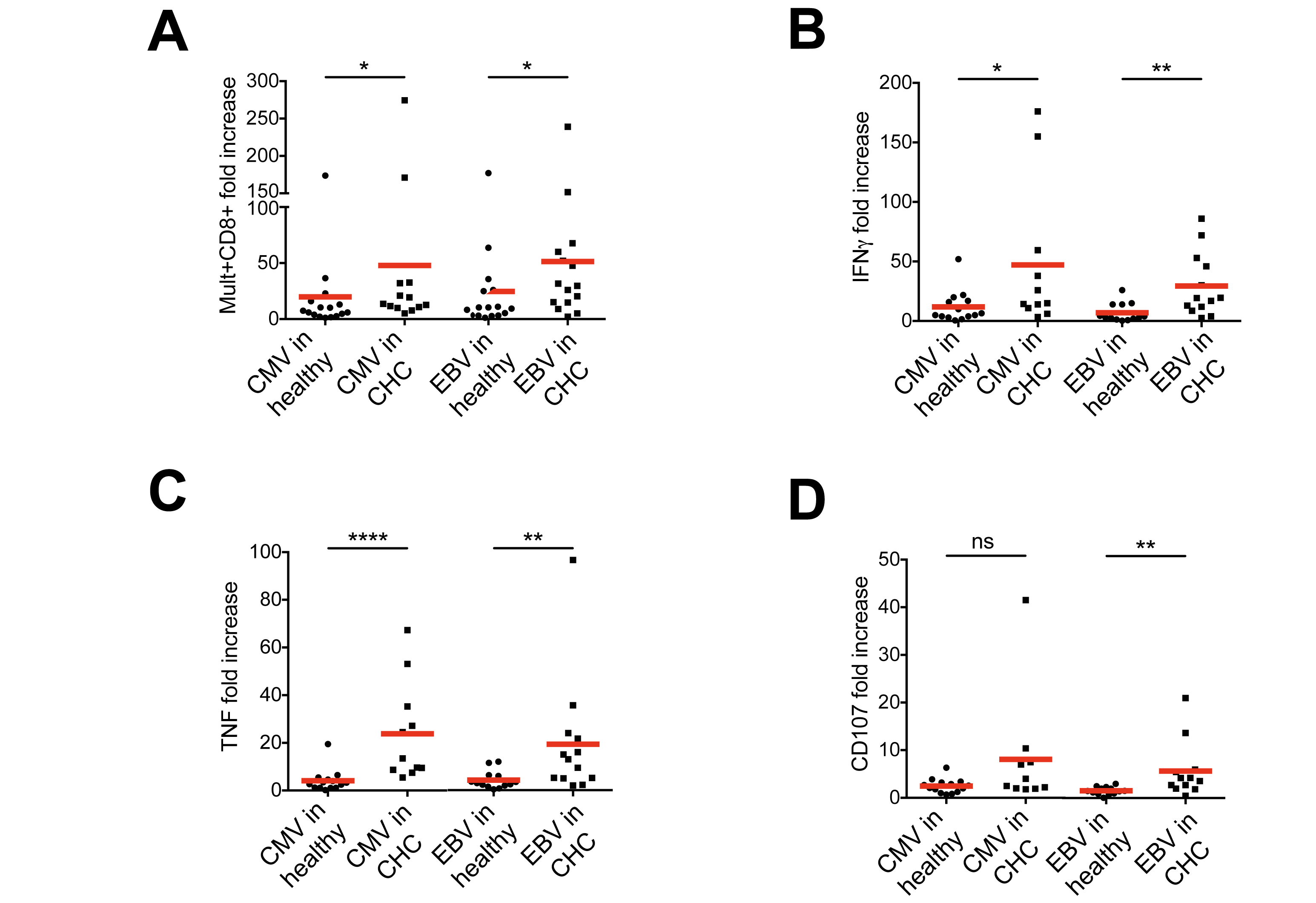

Supplement: Supplementary file 4 [file image_3.tif]

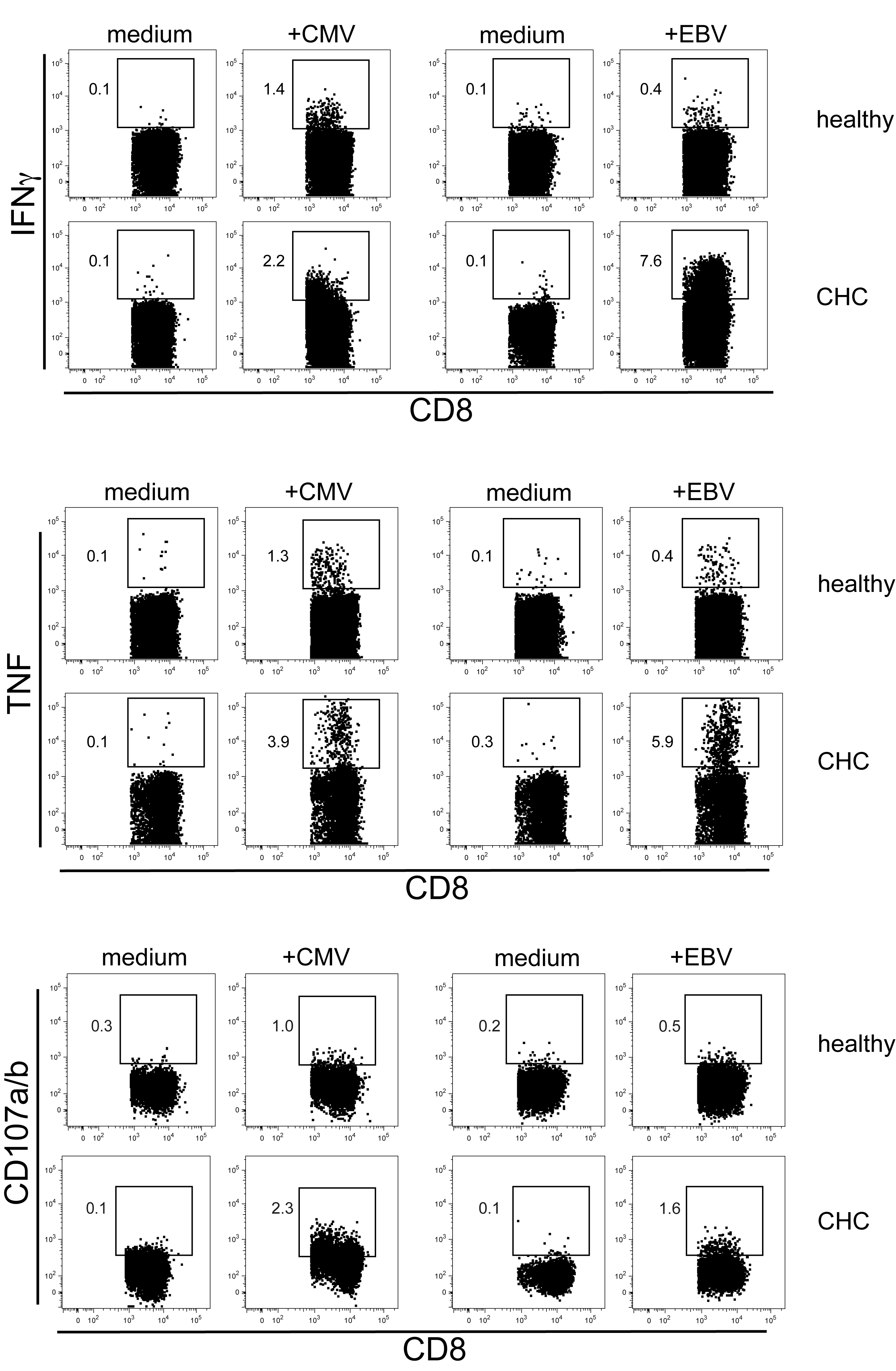

Supplement: Supplementary file 5 [file image_4.tif]
